# Association of irritable bowel syndrome with depression, anxiety, stress, and sociodemographic factors in Saudi Arabia: a cross-sectional survey

**DOI:** 10.3389/fpubh.2026.1866985

**Published:** 2026-08-07

**Authors:** Mukhtiar Baig, Mustafa Ahmed Alhammad, Waleed Ahmed Alghamdi, Shahad Abduljalil Abualhamael, Ali Hassan Jaber Alzahrani, Abdulrahman Abdulmohsen Alharbi, Khadijah Jassim Alhassan, Dhafer Mohammed Alsagoor, Bandar Halil Alyoubi, Turki Akram Alkaabi, Ammar Abdulkhaliq Alshuaibi, Rehaf Fahad AlHarbi, Ahmed Ibrahim Alkhurayyif

**Affiliations:** 1 Department of Clinical Biochemistry, Faculty of Medicine in Rabigh, King Abdulaziz University, Jeddah, Saudi Arabia; 2 Department of Medicine, King Abdulaziz University, Rabigh, Saudi Arabia; 3 Department of Psychiatry, Faculty of Medicine, King Abdulaziz University, Jeddah, Saudi Arabia; 4 Department of Internal Medicine, Faculty of Medicine in Rabigh, King Abdulaziz University, Jeddah, Saudi Arabia; 5 Faculty of Medicine in Rabigh, King Abdulaziz University, Rabigh, Saudi Arabia; 6 Department of Obstetrics and Gnecology, Dar Afia Hospital, Dammam, Saudi Arabia; 7 Maternity and Children Hospital, Najran, Saudi Arabia; 8 Faculty of Medicine in Rabigh, King Abdulaziz University, Jeddah, Saudi Arabia; 9 Faculty of Medicine, Almaarefa University, Riyadh, Saudi Arabia

**Keywords:** anxiety, associated factors, depression, irritable bowel syndrome, lifestyle impact, Rome IV criteria, stress

## Abstract

**Background:**

Irritable bowel syndrome (IBS) is a chronic functional bowel disease and a common cause of gastroenterological referrals. This cross-sectional survey estimated the prevalence of Rome IV-defined IBS symptoms among adults residing in Saudi Arabia and examined their associations with sociodemographic characteristics, and self-reported symptoms of depression, anxiety, and stress.

**Methodology:**

A cross-sectional online survey was conducted from August 2024 to March 2025 among adults aged 18 years or above residing in the five regions of Saudi Arabia. It was coordinated by the Faculty of Medicine, King Abdulaziz University, Rabigh, Saudi Arabia. Participants were recruited through electronic platforms, and data were collected using a self-administered questionnaire that included sociodemographic variables, Rome IV symptom questions with the Bristol Stool Form Scale, and Arabic version of the Depression, Anxiety, and Stress Scale-21 (DASS-21).

**Results:**

A total of 1,553 participants completed the survey. Overall, 373 participants (24%) met the Rome IV symptom-based criteria for IBS. Participants with IBS symptoms had significantly higher mean DASS-21 depression, anxiety, and stress scores than participants without IBS symptoms. IBS symptoms were more common among participants aged 25–50 years, married, living in the Eastern region, and individuals with master's degrees. Among participants who met the Rome IV criteria, all reported recurrent abdominal pain at least once weekly over the past 3 months, and 255 (68.4%) reported that their abdominal pain was always related to time of defecation. A large number of participants (322, 86.3%) who met the Rome IV symptom-based criteria for IBS reported a moderate impact of IBS on their lifestyle. Based on the Bristol Stool Form Scale, 166 participants (44.5%) were classified as IBS-D (diarrhea), 129 (34.6%) as IBS-C (constipation), and 78 (20.9%) as IBS-M (mixed). In multivariable logistic regression, age, education level, and depression, anxiety, and stress symptom categories were statistically associated with IBS symptoms.

**Conclusion:**

In this online cross-sectional survey, 24% of respondents met symptom-based Rome IV criteria for IBS. IBS symptoms were associated with higher self-reported depression, anxiety, and stress scores and selected sociodemographic factors. Longitudinal and clinical studies are needed to clarify temporality and inform integrated gastrointestinal and psychological care.

## 1 Introduction

Irritable bowel syndrome (IBS) is a chronic functional disorder of the gastrointestinal tract and is one of the most commonly diagnosed disorders in gastroenterology clinics (1). Its exact etiopathogenesis is not fully known; however, altered gut motility, visceral hypersensitivity, microbiota-related mechanisms, and psychological distress have all been implicated.

Psychological and social variables may affect gastrointestinal function, symptom perception, illness behavior, and health-seeking patterns (2). Depression, anxiety, and stress are therefore clinically important in IBS because they may intensify symptom burden and impair daily functioning. Worldwide, 10–20% of adults experience symptoms of IBS at some point in their lives, with onset commonly occurring in early adulthood and a higher prevalence reported in women (3). A study using the Rome IV criteria found an IBS prevalence of 16%, with a higher prevalence in females than males (4).

IBS is an episodic disorder, and symptoms can vary across individuals and over time. Common symptoms include abdominal pain, bloating, and alternating episodes of diarrhea and constipation (5). A review study reported that the commencement and seriousness of symptoms can depend on many factors, such as individual characteristics, life experiences, stressors, anxiety, depression, smoking, and eating habits (3). IBS is a highly challenging disorder due to a lack of any laboratory tests, imaging, or invasive procedures that help to identify the presence of the disorder; instead, it is primarily a clinically diagnosed disorder based on the patient's history and examination (5). Diagnosis is based on symptom criteria, especially the Rome IV criteria, which emphasize recurrent abdominal pain for at least 1 day per week during the previous 3 months, associated with pain during defecation, change in the stool frequency or stool form (6).

Extensive evidence supports an association between psychological symptoms and GI disorders, particularly IBS (7). Individuals with IBS usually have depressive and anxiety symptoms (8). The autonomic nervous system (ANS) and stress-responsive neuroendocrine pathways influence GIT motility and sensitivity and may contribute to the link between psychological distress and IBS symptoms (9). The prevalence of psychiatric conditions in IBS ranges from 44% to 84% (10). A Saudi study showed a high prevalence of depression (44.1 %) among patients with IBS (11). A better understanding of these psychological aspects is important for developing patient-centered management approaches.

Several studies from Saudi Arabia have examined IBS prevalence; however, evidence on Rome IV symptom-based IBS and its association with depression, anxiety, stress, and sociodemographic characteristics among adults residing across the five Saudi regions remains limited. Therefore, this study aimed to estimate the prevalence of Rome IV-defined IBS symptoms among adults residing in Saudi Arabia and to examine their associations with sociodemographic factors and self-reported depression, anxiety, and stress symptoms.

## 2 Methods

### 2.1 Study design

The present cross-sectional online survey was coordinated by the Faculty of Medicine, King Abdulaziz University, Rabigh, Saudi Arabia, and conducted between August 2024 and March 2025 among adults residing in Saudi Arabia. Approval for the research protocol was obtained from the King Abdulaziz University Regional Research and Ethics Committee (Reference No. 444-21). Electronic informed consent was obtained from all participants before questionnaire completion, and the confidentiality of the participants was maintained in the research. The research was performed in accordance with the ethical principles outlined in the Declaration of Helsinki.

### 2.2 Inclusion and exclusion criteria

In the present study, adult individuals from all genders (age 18 years and above), and currently residing in Saudi Arabia, including Saudi nationals and non-Saudi residents from all five regions, were eligible for inclusion. Participants who declined consent, were younger than 18 years, lived outside Saudi Arabia, or reported chronic diseases that could confound gastrointestinal symptoms, such as diabetes mellitus, asthma, tuberculosis, cancer, or other major chronic illnesses, were excluded.

### 2.3 Sampling and sample size

The sample size was calculated using the sample size calculator (Raosoft Inc.). We used a 95 percent confidence interval (CI), a 5 percent margin of error, an approximate response rate of 50 percent, and a total population of Saudi Arabia aged >18 years, estimated at 21,600,000. The minimum required sample size was 385. We collected 1,553 responses to improve the precision of the prevalence estimate and subgroup analyses.

### 2.4 Study questionnaire

Data were collected through a self-administered electronic questionnaire distributed via social media groups and other online communication channels commonly used in Saudi Arabia, including WhatsApp, Facebook, Instagram, and TikTok. The survey included three sections. The first section included sociodemographic variables, including gender, age, body weight and height, marital status, nationality, region, and level of education. The questions about IBS were asked in the second section, created using the “Rome IV criteria and the Bristol Stool Form Scale (BSFS)” (12–14). The third section assessed depression, anxiety, and stress symptoms using the Depression, Anxiety, Stress Scale-21(DASS-21) (15). To reduce duplicate responses, the electronic survey settings were configured to limit repeated submissions where possible, and the dataset was screened for incomplete or duplicate entries before analysis.

Participants were categorized as having IBS symptoms when their responses fulfilled the Rome IV symptoms criteria. The criteria require recurrent abdominal pain, on average, at least 1 day per week during the past 3 months, associated with two or more of the following: (a) defecation-related pain, (b) changes in stool frequency, or (c) changes in stool form. The criteria require that these manifestations have existed over the preceding 3-month period, and that the symptoms have emerged at least half a year before the diagnosis. The BSFS has seven types of stools (12). The chart of stool type was uploaded with the questionnaire, and participants were asked to select the type of stool. IBS subtype classification was based on the BSFS responses: IBS-D for predominant loose/watery stools, IBS-C for predominant hard/lumpy stools, and IBS-M for both constipation and diarrhea patterns. Because the study used an online questionnaire, the term ‘IBS symptoms' or ‘participants meeting Rome IV criteria' is used instead of clinician-confirmed IBS diagnosis. DASS-21 scores reflect self-reported symptom severity categories and are not equivalent to clinical psychiatric diagnoses.

The questionnaire used in the study was first translated into Arabic and then back into English. The language versions were reviewed by two bilingual professionals, and the necessary changes were made. They also evaluated the content and face validity of the instrument. Before full deployment, a pilot study with 50 participants was conducted to determine the comprehensibility. The questionnaire was subsequently revised after expert consultation and pilot study findings. The Arabic version of the DASS-21 has been used in Saudi Arabia and is considered a reliable, validated screening tool; however, it is used here only to classify symptom severity, not to diagnose psychiatric disorders.

### 2.5 Statistical analysis

For statistical analysis, SPSS version 21 was used. Categorical variables were represented as frequencies and percentages, and numerical variables as means and standard deviations. Chi-square test was used for the comparison of categorical data, and the independent sample “*t*” test was used for the comparison of continuous data. Binary logistic regression analysis was performed to investigate factors associated with IBS symptoms. The final logistic regression model fitted the data reasonably well. The non-significant Hosmer–Lemeshow test, χ^2^ (8) = 6.514, *p* = 0.590, indicated that the predicted and observed outcomes did not differ significantly. The pseudo-R^2^ values suggested moderate explanatory power, with Cox & Snell R^2^ = 0.249, Nagelkerke R^2^ = 0.373, and McFadden pseudo-R^2^ = 0.260. Missing data were handled by complete-case analysis; incomplete questionnaires and duplicate entries identified during data screening were excluded before final analysis. A *p*-value less than 0.05 was considered statistically significant.

## 3 Results

A total of 1,553 participants completed the survey [female, 956 (61.6%); male, 597 (38.4%)], and the IBS prevalence was 24% (*n* = 373). Overall, 373 participants (24.0%) met the Rome IV symptom-based criteria for IBS. Among participants, 701 (45.1%), 741 (47.7%), and 111 (7.1%) were in the age groups < 25, 25–50, and >50 years, respectively. Most participants had normal weight (43.5%), followed by overweight (27.9%), obesity (19.0%), and underweight (9.6%). Participants meeting IBS criteria had higher proportions of DASS-21 depression, anxiety, and stress symptom categories than participants not meeting IBS criteria (*p* < 0.001). IBS symptoms were more frequent among participants aged 25–50 years, married participants, those residing in the Eastern region, and those with postgraduate education (Table 1).

**Table 1:** Comparison of variables among participants with and without Rome IV-defined IBS symptoms.

Variables	Categories	Total (*n* = 1,553)	No IBS symptoms (*n* = 1,180, 76%)	IBS symptoms (*n* = 373, 24%)	^*^P–value
Age	< 25	701 (45.1%)	640 (54.2%)	61 (16.4%)	< .001
	25–50	741 (47.7%)	461 (39.1%)	280 (75.1%)	
	> 50	111 (7.1%)	79 (6.7%)	32 (8.6%)	
Gender	Female	956 (61.6%)	723 (61.3%)	233 (62.5%)	0.679
	Male	597 (38.4%)	457 (38.7%)	140 (37.5%)	
BMI	Underweight (< 18.5)	149 (9.6%)	111 (9.4%)	38 (10.2%)	0.140
	Normal weight (18.5–24.9)	676 (43.5%)	531 (45.0%)	145 (38.9%)	
	Overweight (25–29.9)	433 (27.9%)	314 (26.6%)	119 (31.9%)	
	Obesity (≥30)	295 (19.0%)	224 (19.0%)	71 (19.0%)	
Marital status	Divorced	44 (2.8%)	29 (2.5%)	15 (4.0%)	< .001
	Married	618 (39.8%)	384 (32.5%)	234 (62.7%)	
	Single	888 (57.2%)	765 (64.8%)	123 (33.0%)	
		57.20%	64.80%	33.00%	
	Widow	3 (0.2%)	2 (0.2%)	1 (0.3%)	
Nationality	Non–Saudi	122 (7.9%)	90 (7.6%)	32 (8.6%)	0.551
	Saudi	1,431(92.1%)	1,090 (92.4%)	341 (91.4%)	
Residence area	Central Region	307 (19.8%)	235 (19.9%)	72 (19.3%)	< .001
	Eastern Region	405 (26.1%)	275 (23.3%)	130 (34.9%)	
	Northern Region	55 (3.5%)	48 (4.1%)	7 (1.9%)	
	Southern Region	390 (25.1%)	298 (25.3%)	92 (24.7%)	
	Western Region	396 (25.5%)	324 (27.5%)	72 (19.3%)	
Educational level	Bachelor Degree	860 (55.4%)	641 (54.3%)	219 (58.7%)	< .001
	Doctorate (PhD)	36 (2.3%)	20 (1.7%)	16 (4.3%)	
	High School	552 (35.5%)	469 (39.7%)	83 (22.3%)	
	Master's Degree	83 (5.3%)	40 (3.4%)	43 (11.5%)	
	No Formal Education	7 (0.5%)	5 (0.4%)	2 (0.5%)	
	Primary School	15 (1.0%)	5 (0.4%)	10 (2.7%)	
Depression	No	485 (31.2%)	451 (38.2%)	34 (9.1%)	< .001
	Yes	1,068(68.8%)	729 (61.8%)	339 (90.9%)	
Anxiety	No	339 (21.8%)	322 (27.3%)	17 (4.6%)	< .001
	Yes	1,214(78.2%)	858 (72.7%)	356 (95.4%)	
Stress	No	1,042(67.1%)	873 (74.0%)	169 (45.3%)	< .001
	Yes	511 (32.9%)	307 (26.0%)	204 (54.7%)	

^*^Chi–square test applied.

All participants meeting IBS criteria reported abdominal pain at least once a week during the previous 3 months, and 255 (68.4%) reported that abdominal pain was always linked to defecation timing. Based on BSFS responses, 166 participants (44.5%), were classified as IBS-D (diarrhea subtype), 129 (34.6%) as IBS-C (constipation subtype), and 78 (20.9%) as IBS-M (mixed subtype) (Table 2).

**Table 2:** Clinical features reported by participants meeting Rome IV–defined IBS symptom criteria (*n* = 373).

Statement	Occurrence	Frequency (%)
In the last 3 months, how many times have you experienced abdomen pain?	It happens rarely (a week may pass without pain)	0 (0)
	Once a week	373(100)
	Never	0 (0)
Has it been 6 months or more since this pain started affecting you	Yes	373(100)
	No	0 (0)
Is this abdominal pain associated with the time around having a defecation either right before, during, or shortly after“	30–50% of the time	118 (31.6)
	Always	255 (68.4)
	Never	0 (0)
When you experience this abdominal pain, your bowel movements either become more frequent or less frequent than usual.”	30–50% of the time	105 (28.2)
	Always	268 (71.8)
	Never	0 (0)
When you experience this abdominal pain, does your stool either become more loose or more hard than usual“	30–50% of the time	103 (27.6)
	Always	270 (72.4)
	Never	0 (0)
Which type of stool usually you pass? Please select from the picture. Type 1 or 2 indicates that you are constipated, while type 6 or 7 indicates that you have diarrhea or sometime constipated sometimes diarrhea.	(6 or 7) Usually diarrhea	166 (44.5)
	(1 or 2) Usually constipated	129 (34.6)
	(Both) constipation and diarrhea	78 (20.9)
How would you describe the intensity of your stomach pain in the past 10 days”	Mild pain	27 (7.2)
	Moderate pain	322(86.3)
	Severe pain	24 (6.4)
Are you currently experiencing (in the past 10 days) bloating in your stomach?	Yes	373(100)
	No bloating	0 (0)
How was the intensity of your stomach bloating in the past 10 days?	Mild bloating	343(92)
	Severe bloating	30 (8)
	None	0 (0)
How much discomfort have you experienced from your bowel functions in the past 10 days¿‘	Mild discomfort	313 (83.9)
	Severe discomfort	55 (14.7)
	No discomfort	5 (1.3)
To what extent have stomach pain, discomfort, or changes in bowel function affected your life in general in the past 10 days?”	Moderate changes	322 (86.3)
	Severe changes	51 (13.7)
	No changes	0 (0)

Participants meeting IBS criteria had significantly higher DASS-21 scores than participants not meeting IBS criteria: depression (9.98 ± 4.45 vs 6.43 ± 4.57; *p* < 0.001), anxiety (11.86 ± 5.09 vs 7.46 ± 5.49; *p* < 0.001), and stress (8.93 ± 5.41 vs 5.26 ± 5.23; *p* < 0.001) (Table 3).

**Table 3:** Comparison of DASS scores among participants with and without Rome IV–defined IBS symptoms.

Disorders	Variables	Mean	Std. deviation	^*^P–value
Depression score	No IBS symptoms	6.43	4.569	< .001
	IBS symptoms	9.98	4.454	
Anxiety score	No IBS symptoms	7.46	5.489	< .001
	IBS symptoms	11.86	5.092	
Stress score	No IBS symptoms	5.26	5.227	< .001
	IBS symptoms	8.93	5.414	

^*^Independent sample “t”–test.

A higher proportion of participants meeting IBS criteria had extremely severe depression, anxiety, and stress symptom categories than participants not meeting IBS criteria (*p* < 0.001) (Table 4). In binary logistic regression age, educational status, and depression, anxiety, and stress symptoms were statistically associated with IBS symptoms (Table 5).

**Table 4:** Comparison of incidence of depression, anxiety and stress severity categories among participants with and without Rome IV IBS symptoms.

Variable	Severity levels	Total (*n* = 1,553)	Rome IV–defined IBS symptoms		^*^P–value
			No (*n* = 1,180)	Yes (*n*=373)	
Depression	Normal	485 (31.2%)	451 (38.2%)	34 (9.1%)	< .001
	Mild	259 (16.7%)	201 (17.0%)	58 (15.5%)	
	Moderate	431 (27.8%)	316 (26.8%)	115 (30.8%)	
	Severe	197 (12.7%)	115 (9.7%)	82 (22.0%)	
	Extremely Severe	181 (11.7%)	97 (8.2%)	84 (22.5%)	
Anxiety	Normal	339 (21.8%)	322 (27.3%)	17 (4.9%)	< .001
	Mild	86 (5.5%)	73 (6.2%)	13 (3.5%)	
	Moderate	315 (20.3%)	254 (21.5%)	61 (16.4%)	
	Severe	172 (11.1%)	137 (11.6%)	35 (9.4%)	
	Extremely Severe	641 (41.3%)	394 (33.4%)	247 (66.2%)	
Stress	Normal	1,042 (67.1%)	873 (74.0%)	169 (45.3%)	< .001
	Mild	118 (7.6%)	72 (6.1%)	46 (12.3%)	
	Moderate	149 (9.6%)	89 (7.5%)	60 (16.1%)	
	Severe	152 (9.8%)	94 (8.0%)	58 (15.5%)	
	Extremely Severe	92 (5.9%)	52 (4.4%)	40 (10.7%)	

^*^Chi–square test applied.

**Table 5:** Binary logistic regression analysis of factors associated with Rome IV–defined IBS symptoms.

Variables	Responses	B	S.E.	Wald	Adjusted OR	95% CI for Adjusted OR	^*^P–value
Age (years)	< 25				1.000		
	25–50	1.708	0.224	58.280	5.518	3.559–8.555	< .001
	>50	1.081	0.331	10.665	2.949	1.541–5.644	0.001
Gender	Male				1.000		
	Female	0.252	0.159	2.507	1.286	0.942–1.756	0.113
BMI (kg/m2)	Underweight (< 18.5)				1.000		
	Normal weight (18.5–24.9)	0.069	0.250	0.075	1.071	0.656–1.747	0.785
	Overweight (25–29.9)	0.304	0.259	1.375	1.355	0.815–2.250	0.241
	Obesity (≥30)	0.038	0.278	0.019	1.039	0.602–1.793	0.891
Marital status	Divorced				1.000		
	Married	0.122	0.381	0.103	1.130	0.535–2.385	0.749
	Single	−0.475	0.401	1.399	0.622	0.283–1.365	0.236
	Widow	0.606	1.737	0.122	1.833	0.061–55.369	0.727
Nationality	Saudi				1.000		
	Non–Saudi	0.074	0.257	0.083	1.077	0.650–1.783	0.773
Residence	Central Region				1.000		
	Eastern Region	0.027	0.213	0.016	1.027	0.676–1.561	0.899
	Northern Region	−0.137	0.472	0.084	0.872	0.346–2.198	0.772
	Southern Region	−0.105	0.218	0.234	0.900	0.587–1.379	0.629
	Western Region	0.086	0.228	0.142	1.090	0.697–1.707	0.705
Educational level	Bachelor degree				1.000		
	Doctorate (PhD)	0.828	0.416	3.954	2.289	1.012–5.178	0.047
	High school	0.022	0.176	0.015	1.022	0.723–1.444	0.902
	Master's degree	0.955	0.282	11.434	2.598	1.494–4.519	< .001
	No formal education	0.487	0.979	0.247	1.627	0.239–11.090	0.619
	Primary school	2.768	0.682	16.495	15.929	4.188–60.581	< .001
Depression	No				1.000		
	Yes	1.340	0.241	30.913	3.820	2.382–6.128	< .001
Anxiety	No				1.000		
	Yes	1.206	0.312	14.934	3.340	1.812–6.158	< .001
Stress	No				1.000		
	Yes	0.753	0.152	24.494	2.123	1.576–2.861	< .001

^*^Binary logistic regression analysis. Dependent variable: meeting Rome IV symptom-based criteria for IBS (1 = yes, 0 = no).

## 4 Discussion

The present study found that 24% of respondents met Rome IV symptom-based criteria for IBS in the study population and markedly higher symptoms in females than in males (61.6% versus 38.4%). These findings are consistent with many other Saudi studies that have reported 9%-40% IBS prevalence (1, 16–21). Almasary et al. (22) reported a pooled IBS prevalence of 20.7% in the Saudi population. Other researchers have also established a higher prevalence of IBS in females than in males (18, 22). Differences between studies may reflect variation in diagnostic criteria, sampling methods, population characteristics, and data-collection procedures. As the present study contained more younger populations (students and young adults), they are prone to more stress-related triggers like academic pressures that can affect the gut–brain axis. In the present study, there was a higher proportion of females and IBS is usually more common among females than males. The causes of gender differences in IBS remain unknown. However, Anbardan et al. claim that the variation of reproductive hormones and their effect on intestinal motility are the cause of female domination (23).

In the present study, IBS status was based on self-reported Rome IV symptoms rather than clinician-confirmed diagnosis; therefore, the findings should be interpreted as symptom-based prevalence among survey respondents.

In the present study, 75% of participants meeting IBS criteria were aged 25–50-year. which showed that younger adults are more prone to developing IBS than older individuals. Similar findings have been reported in severalstudies that observed higher IBS symptom prevalence among younger adults (1, 19, 24, 25). However, the online recruitment method may have increased the participation of younger and more digitally active individuals; therefore, age-related findings require cautious interpretation.

The observed high prevalence in the 25–50year age group may reflect greater exposure to academic, occupational, financial, and family related stressors. All of which can influence the gut-brain axis. It may also show greater symptom recognition and willingness to respond to online surveys among younger adults. Consequently, these findings should be viewed as associations within the surveyed sample rather than evidence that age causes IBS.

The current study observed no significant association between IBS symptoms and BMI. There are inconsistent reports about the direct associations between BMI and IBS symptoms. Similar to our results, another Saudi study reported no association between IBS and BMI (21). This finding supports the possibility that psychological and functional gut-brain mechanisms may be more relevant than adiposity in this sample, although dietary habits, physical activity, and sleep quality were not comprehensively assessed.

The present study showed that participants meeting IBS criteria had significantly higher rates of depression, anxiety, and stress scores than those not meeting IBS criteria. These findings align with several Saudi studies reporting associations between IBS and psychological distress (2, 16, 20, 26–29). International studies have also reported that IBS symptoms are more common among people with higher levels of anxiety or depression than in their peers (25, 30, 31). A Saudi meta-analysis highlighted that psychological factors, specifically anxiety, depression, and stress, are significant contributors to IBS prevalence (overall pooled prevalence ≈20.7 %) (22). The association between psychological distress and IBS symptoms is biologically plausible through the gut-brain axis. Stress-related neuroendocrine and autonomic changes may influence gut motility, visceral sensitivity, and symptom perception (12, 13, 32, 33). Conversely, the symptoms of chronic IBS, which are pain and bloating along with unpredictable bowel movements, may increase psychological distress and seriously impair daily functioning. These also induce an individual's social withdrawal and affect work functioning. These adverse effects can trigger or further aggravate the symptoms of anxiety and depression, and thus, IBS and psychological distress often develop a vicious cycle. So, the relationship may be bidirectional, and longitudinal studies are required to clarify temporality.

In the present study, IBS symptoms were more frequent among respondents from the Eastern region. Previous Saudi studies have reported inconsistent regional patterns, with higher rates reported in different regions, including Central, Eastern, Northern, and Western areas (25, 34, 35). These differences may reflect sampling variation, urbanization, lifestyle factors, health awareness, or unequal online survey reach. Therefore, regional findings should be interpreted with caution and not generalized without probability-based regional sampling.

IBS symptoms were also more frequent among participants with postgraduate education. This may relate to greater health awareness, symptom recognition, academic or occupational stress, or sampling characteristics. Other studies have reported variable associations between education and IBS (5, 17, 36–38). Therefore, educational differences should be interpreted as sample-specific associations rather than causal effects.

Regarding IBS subtypes, IBS-D was the most frequent subtype in this sample (44.5%), followed by IBS-C (34.6%) and IBS-M (20.9%). Other Saudi studies have reported different subtype distributions, with some identifying IBS-M as the most common subtype (1, 24). These differences may reflect variation in stool form assessment, symptom fluctuation over time, treatment exposure, and survey methodology.

Many participants meeting IBS criteria reportedthat bowel symptoms affected their daily life. Although the present study asked about the lifestyle impact, it did not use a validated quality of life instrument... Therefore, the findings should be interpreted as self-reported lifestyle impact rather than formal health-related quality-of-life measurement. Our finding is consistent with previous Saudi and international studies that have shown that IBS can impair emotional, dietary, social, and occupational functioning (35, 39, 40).

A number of reasonable assumptions can be made about why IBS affects daily life. The symptom complex associated with IBS that encompasses abdominal pain, abdominal distension, diarrhea and/or constipation is variable and unpredictable, thus causing reluctance in affected people to attend social events, make long journeys or perform daily occupational roles. Also, patients with IBS may impose restrictive dietary regimes and anxiety around food consumption, which further reduces daily functioning. There is a need for IBS patients to constantly monitor their food intake, which increases stress and decreases eating enjoyment. Such things collectively affect their satisfaction with life. Future studies should include validated quality-of-life scales to quantify this burden more accurately.

These findings support integrated care approaches that consider both gastrointestinal symptoms and psychological distress. Public education and awareness in primary care may help individuals recognize IBS symptoms, reduce stigma, and seek appropriate care. However, interventions should be guided by clinically confirmed studies and longitudinal evidence rather than cross-sectional associations alone.

### 4.1 Limitations

The current study has several limitations. First, the cross-sectional design prevents conclusions about causality or temporality. Second, data were collected via a self-reported online questionnaire, which may be affected by recall, response, and selection biases. Third, although Rome IV criteria and DASS-21 were used, but IBS status and psychological symptom categories were not confirmed by clinical interview or medical history review. Fourth, the survey did not comprehensively assess alarm symptoms or exclude all individuals with possible alarm features, which may have resulted in misclassification. Fifth, several potential confounders, including diet, sleep quality, physical activity, family history, medication use, and detailed comorbid conditions, were not fully assessed. Sixth, the study did not include a validated health-related quality-of-life scale. Finally, although responses were obtained from all Saudi regions, online non-probability recruitment limits generalizability. Future longitudinal and clinically confirmed studies using probability sampling and validated quality-of-life measures are recommended.

## 5 Conclusion

In this survey, 24% of respondents met symptom-based Rome IV criteria for IBS. IBS symptoms were associated with higher DASS-21 depression, anxiety, and stress scores and with selected sociodemographic factors. These results highlight the importance of considering psychological distress when evaluating individuals with IBS symptoms. Longitudinal, clinically confirmed studies are needed to clarify temporality and guide integrated gastrointestinal and mental health interventions in Saudi Arabia.

## Data Availability

The raw data supporting the conclusions of this article will be made available by the authors, without undue reservation.

## References

[1] Alharbi MH Alhazmi AH Ujaimi MH Alsarei M Alafifi MM Baalaraj FS . The prevalence of irritable bowel syndrome and its relation to psychiatric disorders among citizens of Makkah Region, Saudi Arabia. Cureus. (2022) 14:e32705. doi: 10.7759/cureus.32705 36545358 PMC9762683

[2] Almadi MK Sabr MS Kofi M Alaboodi T Al Sayari TA Sabr M. Epidemiology and risk factors of irritable bowel syndrome in the Saudi population: a systematic review. Cureus. (2025) 17:e79974. doi: 10.7759/cureus.79974 40182386 PMC11966369

[3] Black CJ Ford AC. Global burden of irritable bowel syndrome: trends, predictions and risk factors. Nat Rev Gastroenterol Hepatol. (2020) 17:473–86. doi: 10.1038/s41575-020-0286-8 32296140

[4] Arishi AM Elmakki EE Hakami OM Alganmy OM Maashi SM Al-Khairat HK . Irritable bowel syndrome: prevalence and risk factors in Jazan Region, Saudi Arabia. Cureus. (2021) 13:e15979. doi: 10.7759/cureus.15979 34336470 PMC8316899

[5] Canavan C West J Card T. The epidemiology of irritable bowel syndrome. Clin Epidemiol. (2014) 6:71–80. doi: 10.2147/CLEP.S40245 24523597 PMC3921083

[6] Palsson OS Whitehead WE Van Tilburg MA Chang L Chey W Crowell MD . Rome IV diagnostic questionnaires and tables for investigators and clinicians. Gastroenterology. (2016). 10.1053/j.gastro.2016.02.014 27144634

[7] Gong W Guo P Li Y Liu L Yan R Liu S . Role of the gut-brain axis in the shared genetic etiology between gastrointestinal tract diseases and psychiatric disorders: a genome-wide pleiotropic analysis. JAMA Psychiatry. (2023) 80:360–70. doi: 10.1001/jamapsychiatry.2022.4974 36753304 PMC9909581

[8] Goodoory VC Mikocka-Walus A Yiannakou Y Houghton LA Black CJ Ford AC. Impact of psychological comorbidity on the prognosis of irritable bowel syndrome. Am J Gastroenterol. (2021) 116:1485–94. doi: 10.14309/ajg.0000000000001247 33840729

[9] Ali HS Ibrahim Y Saati AA Esam-Eldin E Al Harbi MI. Prevalence of irritable bowel syndrome and its relation to self-esteem, depression, and quality of life of female students in health-related faculties at Umm Al-Qura University. J Am Sci. (2016) 12:91–102.

[10] Banerjee A Sarkhel S Sarkar R Dhali GK. Anxiety and Depression in Irritable Bowel Syndrome. Indian J Psychol Med. (2017) 39:741–5. doi: 10.4103/IJPSYM.IJPSYM_46_17 29284804 PMC5733421

[11] Aljammaz KI Alrashed AA Alzwaid AA. Irritable bowel syndrome: epidemiology and risk factors in the adult Saudi population of the central region. Niger J Clin Pract. (2020) 23:1414–1418. doi: 10.4103/njcp.njcp_382_19 33047699

[12] Lacy BE Mearin F Chang L Chey WD Lembo AJ Simren M . Bowel disorders. Gastroenterology. (2016) 150:1393–407. doi: 10.1053/j.gastro.2016.02.031 27144627

[13] Drossman DA Hasler WL. Rome IV-functional GI disorders: disorders of gut-brain interaction. Gastroenterology. (2016) 150:1257–61. doi: 10.1053/j.gastro.2016.03.035 27147121

[14] Lewis SJ Heaton KW. Stool form scale as a useful guide to intestinal transit time. Scand J Gastroenterol. (1997) 32:920–4. doi: 10.3109/00365529709011203 9299672

[15] Lovibond SH. Manual for the Depression Anxiety Stress Scales. Sydney: Psychology Foundation of Australia. (1995). doi: 10.1037/t01004-000

[16] El-Fetoh NM Abd El-Mawgod MM Mohammed NA Alruwaili HS Alanazi EO. Irritable bowel syndrome among medical and non-medical Northern Border University students, Kingdom of Saudi Arabia: a cross-sectional study. Open J Gastroenterol. (2016) 6:188–95. doi: 10.4236/ojgas.2016.66024

[17] AlAmeel T Roth LS Al Sulais E. The prevalence of irritable bowel syndrome among board-certified medical doctors in Saudi Arabia: a cross-sectional study. J Can Assoc Gastroenterol. (2020) 3:e32–6. doi: 10.1093/jcag/gwz020 33241184 PMC7678731

[18] AlKhalifah MI Al-Aql AM Al-Mutairi MS Alnuqaydan SA Al-Wehaibi AS AlJurayyed AM . Prevalence of irritable bowel syndrome among Qassim school teachers, and its impact on their performance and life duties. Saudi Med J. (2016) 37:817. doi: 10.15537/smj.2016.7.15043 27381548 PMC5018652

[19] Ibrahim NK Battarjee WF Almehmadi SA. Prevalence and predictors of irritable bowel syndrome among medical students and interns in King Abdulaziz University, Jeddah. Libyan J Med. (2013) 8:21287. doi: 10.3402/ljm.v8i0.21287 24054184 PMC3779356

[20] AlButaysh OF AlQuraini AA Almukhaitah AA Alahmdi YM Alharbi FS. Epidemiology of irritable bowel syndrome and its associated factors in Saudi undergraduate students. Saudi J Gastroenterol. (2020) 26:89–93. doi: 10.4103/sjg.SJG_459_19 32031159 PMC7279070

[21] Almuzaini AS Almuzaini R Alsaleem HN Alsuhaibani A Alsohaibani A Alwehaibi R . Prevalence and associated risk factors of irritable bowel syndrome among general population: a cross-sectional study in Qassim Region, Saudi Arabia. Cureus. (2024) 16:e57493. doi: 10.7759/cureus.57493 38707106 PMC11068117

[22] Almasary M Alkhalifah KM Alotaibi SH Elhefny M Alabssi H Alaklabi SS . Prevalence of irritable bowel syndrome in Saudi Arabia: a systematic review and meta-analysis. Cureus. (2023) 15:e45357. doi: 10.7759/cureus.45357 37849586 PMC10577611

[23] Anbardan SJ Daryani NE Fereshtehnejad SM Vakili ST Keramati MR Ajdarkosh H. Gender role in irritable bowel syndrome: a comparison of irritable bowel syndrome module (Rome III) between male and female patients. J Neurogastroenterol Motil. (2012) 18:70. doi: 10.5056/jnm.2012.18.1.70 22323990 PMC3271257

[24] Makkawy EA Abdulaal IE Kalaji FR Makkawi M Alsindi N Makkawy EA. Prevalence, risk factors, and management of irritable bowel syndrome in Saudi Arabia: a systematic review. Cureus. (2023) 15:e47440. doi: 10.7759/cureus.47440 38021554 PMC10658819

[25] Alharbi SH Alateeq FA Alshammari KI Ahmed HG. IBS common features among Northern Saudi population according to Rome IV criteria. AIMS Med Sci. (2019) 6:148–57. doi: 10.3934/medsci.2019.2.148

[26] Ibrahim NK Al-Jamhoor S Ashor N Alsulami AA Bukhari DA Al-Bloshi AM . Irritable bowel syndrome among paramedical students, King Abdulaziz University, Jeddah, Saudi Arabia. J Adv Med Med Res. (2018) 25:1–9. doi: 10.9734/JAMMR/2018/39233

[27] Alhammadi NA Bedywi RM Shawkhan RA Aljari AA Asiri SA Al Hamdan JA . Migraine and irritable bowel syndrome among the general population in Aseer region. Cureus. (2023) 15:e45047. doi: 10.7759/cureus.45047 37829989 PMC10566572

[28] Alshaikh AA Alamri SM Riaz F Mahmood SE Shlwan MA Almuidh FN . Exploring the burden of irritable bowel syndrome among university students in Saudi Arabia: a study on prevalence, psychological associations, and well-being. Medicine. (2024) 103:e38099. doi: 10.1097/MD.0000000000038099 38728477 PMC11081599

[29] Alharbi MS Alharbi RM Almutairi MQ Khan FH Alsulami EA Alenezi AM . Anxiety and depression among patients with irritable bowel syndrome in Hail, Saudi Arabia: towards a better understanding of the psychological role. Cureus. (2024) 16:e73290. doi: 10.7759/cureus.73290 39655118 PMC11625521

[30] Sibelli A Chalder T Everitt H Workman P Windgassen S Moss-Morris R . Systematic review with meta-analysis of the role of anxiety and depression in irritable bowel syndrome onset. Psychol Med. (2016) 46:3065–80. doi: 10.1017/S0033291716001987 27605134

[31] Midenfjord I Polster A Sjövall H Törnblom H Simrén M. Anxiety and depression in irritable bowel syndrome: exploring the interaction with other symptoms and pathophysiology using multivariate analyses. Neurogastroenterol Motil. (2019) 31:e13619. doi: 10.1111/nmo.13619 31056802

[32] Mayer EA Tillisch K Gupta A. Gut/brain axis and the microbiota. J Clin Invest. (2015) 125:926–38. doi: 10.1172/JCI76304 25689247 PMC4362231

[33] Qin HY Cheng CW Tang XD Bian ZX. Impact of psychological stress on irritable bowel syndrome. World J Gastroenterol. (2014) 20:14126. doi: 10.3748/wjg.v20.i39.14126 25339801 PMC4202343

[34] Alhazmi A Darraj H Abdali H Hakami SM Alatiyyah A Dalak M . Anxiety-related factors associated with symptom severity in irritable bowel syndrome in Jazan, Saudi Arabia. Cureus. (2024) 16:e53549. doi: 10.7759/cureus.53549 38445128 PMC10913131

[35] Aljahdli ES Badroun F Mushaeb HH Aljohani R Albisher S Basalaim L . Effects of irritable bowel syndrome on the health-related quality of life among the Saudi population. Saudi J Gastroenterol. (2024) 30:37–44. doi: 10.4103/sjg.sjg_107_23 37357494 PMC10852141

[36] Alkalash SH Almagadi RA Alamri SM Al-Amri LA Al-Amri MA Al-Amri JM . Prevalence and risk factors of irritable bowel syndrome among adults in Al-Qunfudah Governorate, Saudi Arabia. Cureus. (2023) 15:e48639. doi: 10.7759/cureus.48639 38090431 PMC10711523

[37] Perveen I Parvin R Saha M Bari MS Huda MN Ghosh MK. Prevalence of irritable bowel syndrome (IBS), migraine and co-existing IBS-migraine in medical students. J Clin Diagn Res. (2016) 10:OC09. doi: 10.7860/JCDR/2016/20900.8832 28050419 PMC5198372

[38] Amin HS Irfan F Karim SI Almeshari SM Aldosari KA Alzahrani AM . The prevalence of irritable bowel syndrome among Saudi population in Riyadh by use of Rome IV criteria and self-reported dietary restriction. Saudi J Gastroenterol. (2021) 27:383–90. doi: 10.4103/sjg.sjg_43_21 34747875 PMC8656325

[39] AlDosari MN Alotaibi RM Algahtani MN Alshammari TS Almziri BF ALOTAIBI R . Assessing the impact of irritable bowel syndrome on quality of life in patients at family medicine and primary health care clinics of the National Guard Health Affairs, Riyadh. Cureus. (2024) 16:e76158. doi: 10.7759/cureus.76158 39717523 PMC11665739

[40] Zhu L Huang D Shi L Liang L Xu T Chang M . Intestinal symptoms and psychological factors jointly affect quality of life of patients with irritable bowel syndrome with diarrhea. Health Qual Life Outcomes. (2015) 13:49. doi: 10.1186/s12955-015-0243-3 25925746 PMC4414422

